# *Pichia occidentalis* volatiles elicit divergent behavioral responses in *Drosophila suzukii* and *Drosophila melanogaster*

**DOI:** 10.1128/aem.01135-26

**Published:** 2026-10-01

**Authors:** Beth Ferguson, James J. Polashock, Tia Hart, Cesar Rodriguez-Saona, Jennifer S. Sun

**Affiliations:** 1 P.E. Marucci Center for Blueberry and Cranberry Research & Extension, Chatsworth, New Jersey, USA; 2 USDA Agricultural Research Service 17123, Chatsworth, New Jersey, USA; 3 Department of Biochemistry and Microbiology, Rutgers University 574031, New Brunswick, New Jersey, USA; Universite de la Reunion, Ste Clotilde, France

**Keywords:** *Pichia occidentalis*, *Drosophila suzukii*, *Drosophila melanogaster*, chemical ecology, chemosensation, olfaction, behavior, insect control, niche adaptation

## Abstract

*Drosophila suzukii* (spotted-wing drosophila) is an invasive pest that infests ripe, market-ready fruit, posing a significant threat to fruit production. To develop alternatives to conventional chemical control, environmentally derived fungi (EF) were evaluated for their potential to modulate *D. suzukii* behavior and viability. *Pichia occidentalis*, isolated from *Drosophila melanogaster* oral and fecal secretions, exhibited species-specific repellency in *D. suzukii*. In *D. suzukii*, exposure to *P. occidentalis* reduced survival, most pronounced in males, and significantly inhibited female oviposition. In contrast, *D. melanogaster* showed no reduction in survival and increased oviposition in the presence of the fungus. Solid-phase microextraction coupled with gas chromatography–mass spectrometry (SPME GC-MS) identified volatile metabolites produced by *P. occidentalis*. Subsequent cafeteria-style assays of individual volatiles revealed that several of these compounds elicited aversive responses in *D. suzukii* while remaining attractive to *D. melanogaster*, highlighting species-specific olfactory effects. Isoamyl butanoate, 2-phenyl-1-propanal, and benzyl alcohol demonstrated consistent repellent activity with superior efficacy compared to established *D. suzukii* repellents (2-pentylfuran, 1-octen-3-ol, and geosmin). Collectively, these findings demonstrate that EF metabolites can differentially influence closely related species and may be leveraged as targeted, behavior-based tools for managing *D. suzukii*.

**IMPORTANCE:**

*Drosophila suzukii* is a major invasive pest that damages fresh fruit by laying eggs in ripening crops, leading to significant agricultural losses. Current control strategies rely heavily on chemical insecticides, which provide incomplete control and can harm non-target organisms. Here, we show that volatile metabolites produced by the environmental yeast *Pichia occidentalis* differentially influence closely related *Drosophila* species, decreasing oviposition and longevity in *D. suzukii* while stimulating oviposition in *D. melanogaster*. This species-specific divergence highlights the potential of microbial volatiles as precision tools for pest management, further supporting a behavior-based approach that selectively disrupts pest reproduction while minimizing ecological impact.

## INTRODUCTION

As climate change and human activity expand the range and adaptability of agricultural pests, strengthening the resilience and efficiency of our agricultural systems has become both a scientific priority and a necessity for global stability (1). Among the most damaging invasive threats to global production of soft-skinned and stone fruits (i.e., blueberries, cherries, raspberries, blackberries, strawberries, and grapes) is *Drosophila suzukii* (Matsumura), commonly known as the spotted-wing drosophila. Unlike most other *Drosophila* species, which target overripe or decaying fruit, *D. suzukii* lays its eggs in ripe, marketable fruit, causing punctures and surface blemishes, and its larvae feed within the fruit, together rendering crops vulnerable to secondary microbial infection and rapid spoilage (2, 3). A key adaptation distinguishing *D. suzukii* from other *Drosophila* species is its serrated ovipositor, which enables females to penetrate intact fruit skin and oviposit in ripening fruit (4), making females the primary drivers of crop damage and the most consequential targets for control. As *D. suzukii* continues to expand its geographic range (3) and cause substantial losses in fruit yield and revenue (5), there is growing interest in sustainable biological control methods that selectively target this pest without disrupting non-target species.

Current management approaches provide only incomplete control. *D. suzukii* is managed primarily through repeated insecticide applications, supplemented by cultural measures such as frequent harvest and sanitation of fallen and culled fruit (6). These approaches offer only partial protection because *D. suzukii* oviposits in intact, ripening fruit. Sanitation directed at overripe and dropped fruit addresses only part of the population (7). Moreover, insecticidal control is transient, with efficacy reduced by rainfall (7). Insecticides act primarily on exposed surfaces and have a limited direct effect on larvae developing within the fruit, reducing larval numbers mainly indirectly through adult mortality (8). Repeated applications also raise concerns about resistance (8). Additional concerns include non-target effects on beneficial insects and residue restrictions that can lead to crop rejection (6, 9). These limitations underscore the need for alternative strategies that exploit species-specific features of *D. suzukii*’s sensory and reproductive biology. Behavior-based tactics such as repellents and oviposition deterrents are increasingly viewed as components of integrated pest management (IPM) for *D. suzukii*, complementing insecticides by reducing reliance on broad-spectrum sprays and their associated resistance and non-target risks (10–12). Semiochemical-based push-pull strategies, in which repellents drive the pest away from a protected crop while attractants concentrate it in traps or sacrificial hosts, are a particularly promising yet underdeveloped avenue for this pest, limited in part by the small number of characterized species-specific cues.

Microbes have been investigated for their ability to inhibit agricultural pests, including *D. suzukii*, by modulating behavioral and physiological pathways. Entomopathogenic fungi (EPF), such as *Metarhizium anisopliae* (Metchnikoff) Sorokin, infect insects by attaching to the cuticle, penetrating the epidermis, and entering the hemocoel (13), and have been shown to emit olfactory cues that lure *D. suzukii* to its demise (13). While EPF can be effective bioinsecticides, their broad host range across many insect taxa raises concerns about non-target effects on native and beneficial insects such as pollinators and natural enemies (14). A more selective alternative may lie in non-pathogenic, environmentally derived fungi (EF) that influence host behavior or physiology through non-lethal mechanisms, such as altering oviposition decisions or reducing reproductive success. Microbes released by competing *Drosophila* species on plant substrates have been shown to repel *D. suzukii* and inhibit oviposition, an effect attributed in part to inherent differences in gut microbiome composition between species (15). This raises the possibility that fungi associated with competitor *Drosophila* may produce cues that *D. suzukii* interprets as signals of unsuitable host substrates (16).

*D. suzukii* relies heavily on its olfactory system to guide host selection. For oviposition, it exhibits a strong preference for fresh, ripening fruit over overripe or decaying substrates (17). Adults also feed on overripe and fermenting fruit and shift between fruit and fermentation cues in response to physiological state and season (18). This preference is largely mediated by volatile organic compounds (VOCs) emitted by fruit and associated plant material (17). Fungal colonization of fruit alters the volatile profile, influencing *D. suzukii* behavior (19). Several fungal-derived volatiles have been shown to attract *D. suzukii* (20, 21), while others can act as repellents and oviposition deterrents (22). For example, *Colletotrichum fioriniae* (Marcelino, Damm, Crous, and Fourie), a fungal pathogen of blueberries, induces a dose-dependent repellent response in *D. suzukii* (22), demonstrating that fungal metabolites can function as aversive cues under specific conditions. Attraction to yeast volatiles varies among species, and some *Pichia* species, such as *P. terricola*, are attractive to *D. suzukii* (21). Others are not: a field study of wild *D. suzukii* responses to microbial volatiles found that *Pichia* sp. cultures trapped very few flies in raspberry plantings despite producing detectable headspace volatiles, suggesting that the cues from this *Pichia* species may be intrinsically poor attractants or may signal unsuitable host substrates (23). A small number of volatile repellents and oviposition deterrents have been characterized for *D. suzukii*, including geosmin, 1-octen-3-ol, and 2-pentylfuran (19, 22, 24), and, more recently, methyl *N,N*-dimethylanthranilate and ethyl propionate (25). To date, little is known about non-pathogenic fungal species that produce repellent volatile cues, and even less about whether such cues can be species-selective between *D. suzukii* and its close relatives. To date, little is known about non-pathogenic fungal species that produce repellent volatile cues, and even less about whether such cues can be species-selective between *D. suzukii* and its close relatives.

*Pichia occidentalis* (Kurtzman) was identified through a serendipitous observation in a *D. suzukii* rearing colony at the P.E. Marucci Center for Blueberry and Cranberry Research, where an unrecoverable colony collapse occurred following invasion by wild *Drosophila melanogaster* (Meigen). During the collapse, a persistent yeast film appeared on the *D. suzukii* diet in association with oral and fecal secretions deposited by the invading *D. melanogaster*. Behavioral observations during the collapse revealed that *D. suzukii* avoided ovipositing in areas where this film was present. The yeast was subsequently isolated from this *D. melanogaster* oral and fecal secretion and identified by sequencing as *P. occidentalis*. Because this isolate emerged from a natural interaction between competing species and was associated with a clear, repeatable behavioral response, it offered a rare opportunity to investigate whether a single environmental fungus could selectively deter *D. suzukii* while remaining compatible with a non-target *Drosophila* species.

This study tests the hypothesis that *P. occidentalis* exerts species-selective effects on *D. suzukii*, modulating both olfactory behavior and reproduction through volatile-mediated signaling, while producing distinct or attenuated effects in *D. melanogaster*. Our discoveries could identify *P. occidentalis*-derived compounds that can serve as the basis for a behavior-based, species-selective approach to *D. suzukii* management.

## RESULTS

### *P. occidentalis* was isolated from *D. melanogaster* oral and fecal secretions

An unrecoverable *D. suzukii* colony collapse coincided with the emergence of a persistent yeast film on the *D. suzukii* diet, which was present in association with oral and fecal secretions deposited by invading *D. melanogaster*. Behavioral observations during the collapse revealed that *D. suzukii* avoided ovipositing in regions of the diet where this film was present. The yeast was subsequently isolated from this *D. melanogaster* oral and fecal secretion, cultured to purity, and identified as *P. occidentalis* by amplification and sequencing of the ITS (accession number PZ471018) and D1–D2 regions of the large-subunit rRNA gene (accession number PZ502810), followed by nucleotide similarity analysis using BLASTN (100% sequence identity to representative *P. occidentalis* sequences in GenBank, e.g., MK262974.1, KM234444.1, KP171596.1, and over 97% identity to other representatives in the database).

### *P. occidentalis* elicits opposing olfactory responses in *D. suzukii* and *D. melanogaster*

**Fig 1:**
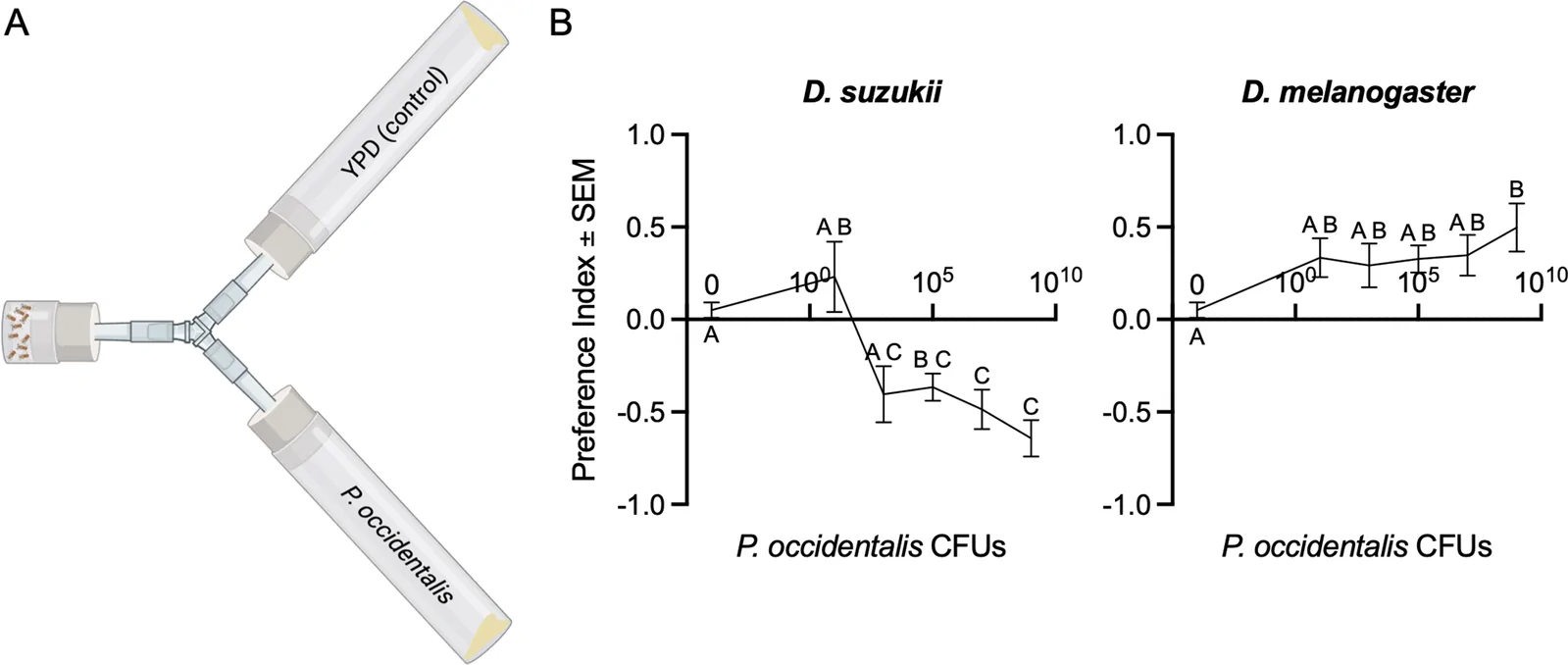
*P. occidentalis* elicits opposing concentration-dependent olfactory responses in *D. suzukii* and *D. melanogaster*. (**A**) Schematic of the two-choice Y-maze olfactory assay. Ten flies (five female, five male, 7 days old) were introduced into the loading vial and allowed 24 h to choose between a trap containing *P. occidentalis* and a trap containing YPD (control). (**B**) Preference index (PI) of *D. suzukii* (left) and *D. melanogaster* (right) in response to *P. occidentalis* across concentrations ranging from 10⁻¹ to 10⁹ CFU/mL. PI = [(# in experimental) − (# in control)] / 10; positive values indicate attraction, negative values indicate avoidance. Bars represent mean ± SEM; *N* = 15 biological replicates per concentration. Differences among concentrations were analyzed using a Kruskal-Wallis test followed by Dunn’s multiple-comparisons test. Different letters above the bars indicate statistically significant differences (*P* < 0.05).

To evaluate whether *P. occidentalis* influences host-seeking behavior, two-choice olfactory assays were conducted across a range of concentrations (10⁻¹ to 10⁹ CFU/mL) (Fig. 1A). *P. occidentalis* elicited avoidance in *D. suzukii* at and above 10^5^ CFU/mL (10^5^ CFU/mL, *P* = 0.0217; 10^7^ CFU/mL, *P* = 0.0030; 10^9^ CFU/mL, *P* = 0.0002), although 10¹ CFU/mL of *P. occidentalis* was modestly attractive (Fig. 1B). In contrast, *P. occidentalis* was attractive to *D. melanogaster*, with a positive preference index across all tested concentrations that reached significance relative to the control at the highest concentration (10^9^ CFU/mL, *P* = 0.0120).

### *P. occidentalis* exposure produces sex-specific effects on *D. suzukii* viability

**Fig 2:**
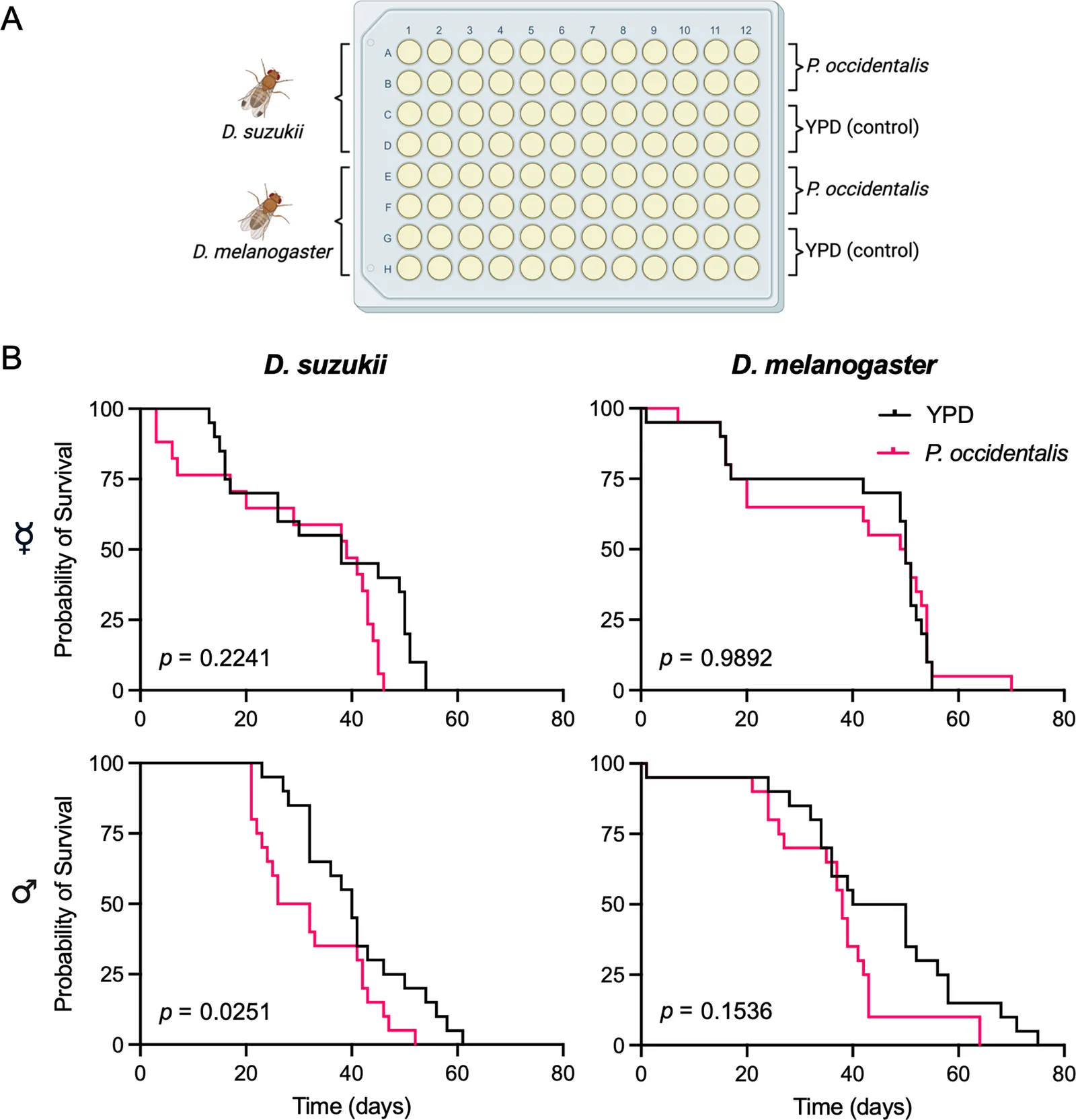
*P. occidentalis* exposure produces sex-specific effects on *D. suzukii* viability. (**A**) Schematic of the 96-well plate adult longevity assay. Individual flies were housed in wells containing standard diet supplemented with either *P. occidentalis* (experimental) or YPD (control) and transferred to fresh treated food daily until the last survivor died. (**B**) Kaplan-Meier survival curves for female and male *D. suzukii* (left) and *D. melanogaster* (right) under continuous exposure to *P. occidentalis* (red) versus YPD-only controls (black). *N* = 24 flies per species, sex, and condition. Differences between survival curves were assessed using the Mantel-Cox (log-rank) test, and hazard ratios with 95% confidence intervals were calculated using a Cox proportional hazards model.

To determine whether the behavioral effects of *P. occidentalis* extended to host viability, adult survival was monitored following continuous exposure to *P. occidentalis*-supplemented diet (Fig. 2A). In *D. suzukii* females, viability was significantly reduced following *P. occidentalis* exposure relative to yeast peptone dextrose (YPD)-only controls. However, the effect was modest and late-emerging: median survival was unchanged (39 vs 38 days), and the difference was significant by the equally weighted log-rank test (*P* = 0.044) but not by the early weighted Gehan-Breslow-Wilcoxon test (*P* = 0.224), indicating the curves diverged mainly late in life (Fig. 2B). *D. suzukii* males, by contrast, showed a stronger and more consistent reduction in viability, significant by both the log-rank (*P* = 0.031) and Gehan-Breslow-Wilcoxon (*P* = 0.025) tests, with median survival reduced from 40 to 29 days and mortality occurring earlier than in YPD controls. Fungus-exposed males had an elevated hazard of death relative to YPD controls (hazard ratio = 0.4592, 95% CI = 0.2268–0.9296). No significant effects on viability were observed in *D. melanogaster* of either sex (females, *P* = 0.732; males, *P* = 0.075). These results indicate that *P. occidentalis* exerts modest, male-biased effects on *D. suzukii* survival and no significant effect on *D. melanogaster*.

### *D. suzukii* avoids *P. occidentalis*-colonized oviposition sites

**Fig 3:**
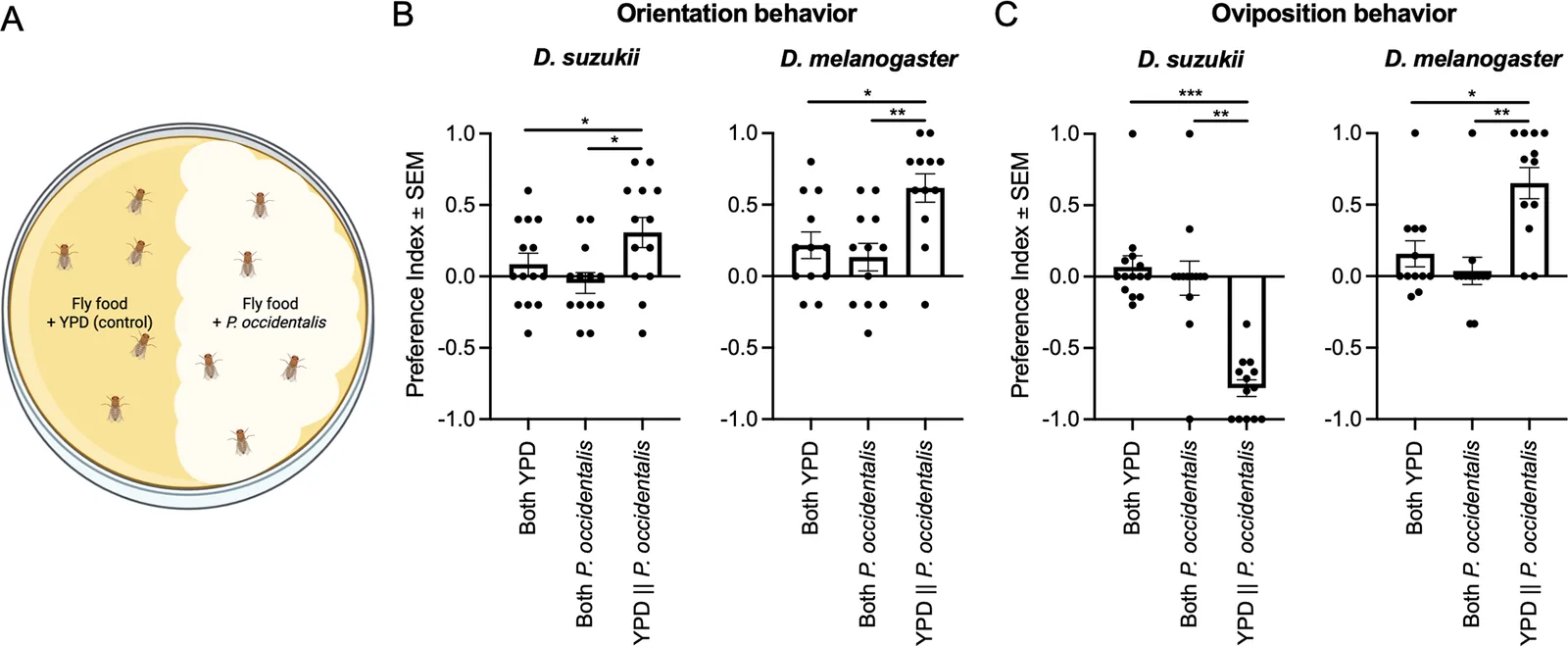
*D. suzukii* avoids *P. occidentalis*-colonized oviposition sites despite preferring fungal-occupied substrates for orientation. (**A**) Schematic of the orientation and oviposition assay. Mated females were placed on plates under three conditions: uniformly inoculated with YPD, uniformly inoculated with *P. occidentalis*, or split plates with YPD on one half and *P. occidentali*s on the other. (**B**) Orientation preference of *D. suzukii* (left) and *D. melanogaste*r (right) measured 15 min after introduction. Preference index (PI) = [(# in experimental) − (# in control)] / 20; positive values indicate attraction, and negative values indicate avoidance. (**C**) Oviposition preference quantified by egg counts after 3 h on each substrate region. Preference index (PI) = [(# in experimental) − (# in control)] / (total # of eggs); positive values indicate attraction, and negative values indicate avoidance. Bars represent mean ± SEM; *N* = 15 independent trials per condition, with 20 mated females per trial. Differences among treatment groups were analyzed using a Kruskal-Wallis test followed by Dunn’s multiple-comparisons test. *P < 0.05, **P < 0.01, ***P < 0.001.

Because the viability data did not support direct lethality as the dominant mode of action, orientation and oviposition assays were performed to test whether *P. occidentalis* influenced reproductive decision-making (Fig. 3A). *P. occidentalis* significantly affected host orientation in both species (Fig. 3B). When assay plates were uniformly inoculated with either YPD or *P. occidentalis*, neither species exhibited a positional preference. When given a choice between YPD and fungal substrates on split plates, however, both *D. suzukii* (*P* = 0.0372) and D. *melanogaster* (*P* = 0.0290) females preferred to occupy the *P. occidentalis*-colonized side, although the magnitude of this orientation preference was modest. This orientation measure is a single positional snapshot recorded 15 min after release, during which flies moved freely across the entire plate and were never confined to either substrate. Orientation, therefore, reflects transient occupancy rather than a committed choice. Orientation in the oviposition assay is not directly comparable to the two-choice Y-maze (Fig. 1), in which flies commit to a single trap arm rapidly (<1 min of assay start) and are then retained there. The weak orientation preference for the fungal side thus does not conflict with the concentration-dependent avoidance measured in the Y-maze, and both readouts are distinct from the oviposition decision.

*P. occidentalis* influenced oviposition behavior differentially between the *Drosophila* species (Fig. 3C). No preference was observed when substrates were uniformly inoculated. However, in two-choice assays, *D. suzukii* females preferentially oviposited on the YPD substrate (*P* = 0.0004), indicating clear avoidance of *P. occidentalis*. In contrast, *D. melanogaster* females preferred to oviposit on the *P. occidentalis*-colonized substrate (*P* = 0.0332). These results decouple host orientation from oviposition decision-making and identify oviposition deterrence, not avoidance of host contact, as the central effect of *P. occidentalis* on *D. suzukii*.

### *P. occidentalis* emits volatile metabolites

To identify the *P. occidentalis* volatile compounds responsible for the observed behavioral effects, we analyzed gas-phase volatiles using solid-phase microextraction (SPME) coupled to gas chromatography–mass spectrometry (GC-MS), a technique widely used to profile microbial VOCs. Twenty-one compounds were identified (Table S1) and classified into six chemical families: aldehydes, alcohols, esters, ketones, acids, and others (Table 1).

**TABLE 1:** Composition of *P. occidentalis* headspace, categorized by chemical class

Aldehydes	Alcohols	Esters	Ketones	Acids	Others
Phenylacetaldehyde	Isoamyl alcohol	Isoamyl formate	Acetophenone	Isovaleric acid	1-octene
Phenylethyl oxyacetaldehyde	Phenylethyl alcohol	Isopropyl butanoate	2-heptanone	2-methylbutanoic acid	(4E)-octene
	6-methylheptan-2-ol	Ethyl isovalerate	2-nonanone	4-pentenoic acid	
	(5Z)-octenol	Isoamyl acetate	2-undecanone		
	2-nonanol				

Headspace sampling with the two SPME fibers yielded largely complementary volatile profiles. The PDMS fiber resolved 11 compounds and the CAR/PDMS fiber 14, of which only four (phenylethyl alcohol, isovaleric acid, 2-heptanone, and 2-nonanone) were common to both, for a combined total of 21 compounds (Table 1). The fibers also differed systematically in which compounds they recovered. The CAR/PDMS fiber preferentially captured earlier-eluting, more volatile constituents (e.g., isopentyl formate, the octenes, and short-chain esters and acids), whereas the PDMS fiber more readily captured later-eluting, less volatile compounds (e.g., acetophenone, 2-nonanol, phenylacetaldehyde, and 2-undecanone), consistent with the known sorption selectivities of the two coatings. Despite these differences, phenylethyl alcohol dominated both profiles, comprising 96.89% of the total peak area with the PDMS fiber and 90.40% with the CAR/PDMS fiber. Sampling with two fibers of distinct selectivity therefore broadened the range of detected volatiles and gave a more complete description of the yeast volatile profile than either fiber alone.

Values are reported as relative percentages of total peak area and describe the relative composition of the headspace rather than absolute compound concentrations. Absolute quantification was not attempted because SPME is a non-exhaustive, equilibrium-based extraction, in which recovery is both compound- and fiber-dependent, and no internal standards or authentic standard calibration curves were included. Peak-area percentages should therefore be interpreted as a semiquantitative profile. For the same reason, each compound’s percentage is normalized within a given fiber and is not directly comparable in absolute terms between the two fibers.

To assemble a panel of candidate volatiles for behavioral evaluation, we drew on the previously published headspace profile of *P. occidentalis* (26). Phenylethyl alcohol and acetophenone were common to both that profile and our own analysis. Moreover, the aromatic alcohol phenylethyl alcohol is the most abundant compound in the headspace of *P. occidentalis*. Phenylethyl alcohol and its acetate ester, phenylethyl acetate, have previously been reported in other yeast species, including *Candida tropicalis* (Castellani) Berkhout, *Saccharomyces cerevisiae* Meyen ex EC Hansen, and *Pichia kudriavzevii* Boidin, Pignal & Besson, alongside ethyl esters of medium-chain fatty acids (27, 28).

### *P. occidentalis* volatiles elicit species-specific repellent responses

**Fig 4:**
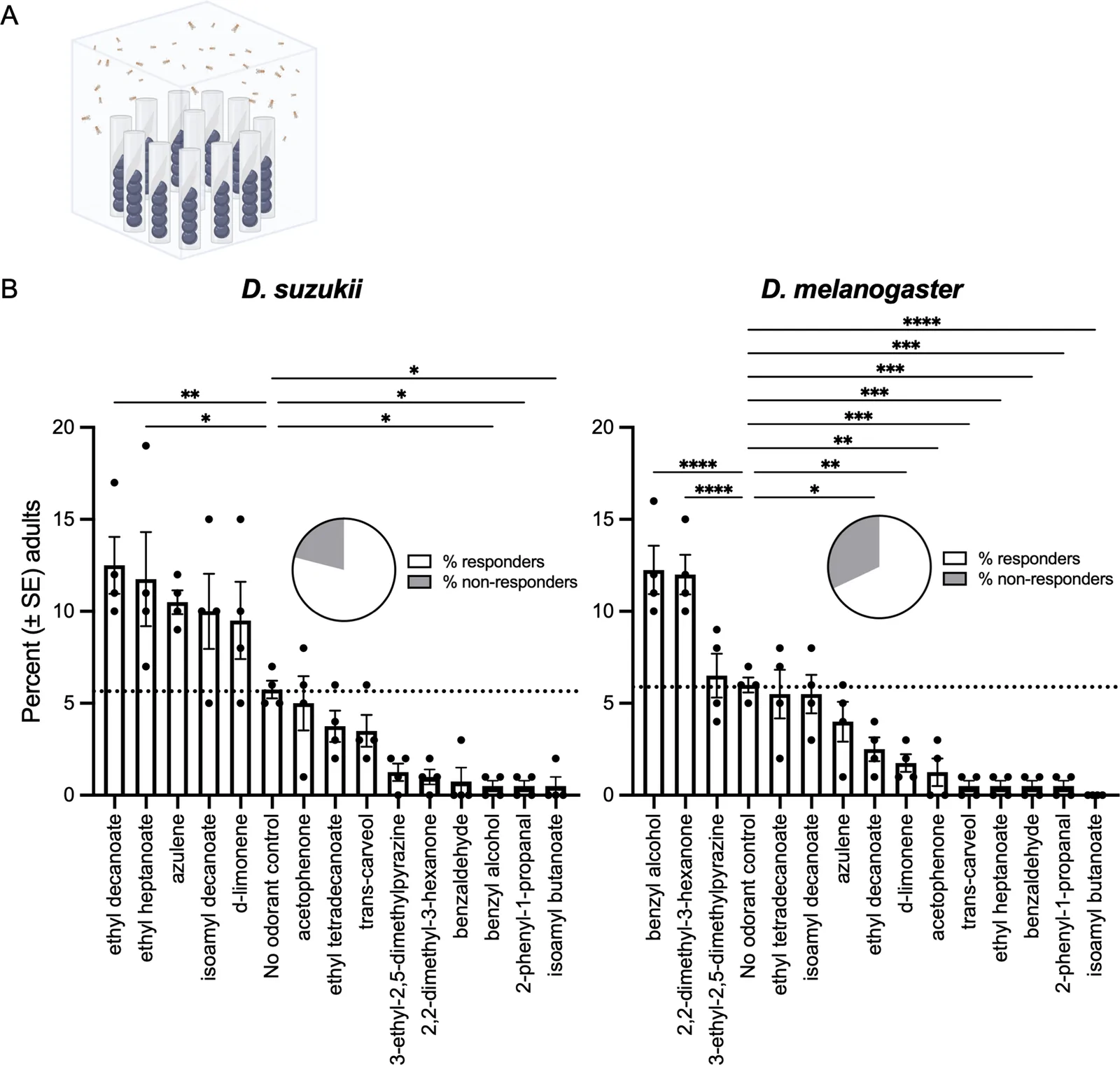
*P. occidentalis*-derived volatiles elicit species-specific behavioral responses in cafeteria-style assays. (**A**) Schematic of the cafeteria-style behavioral assay. Each 14″ × 14″ × 14″ netted cage contained traps arranged equidistantly around a central water source, with control traps containing sterilized blueberries alone and experimental traps containing blueberries plus one *P. occidentalis*-derived volatile (50 µL neat). Three hundred adult flies (1:1 male:female ratio) were released per cage following 2 h of starvation. (**B**) Percent of *D. suzukii* (left) and *D. melanogaster* (right) flies captured per trap after 24 h of exposure to individual volatile compounds, calculated as the number of flies in each trap divided by the 300 flies released per cage. Bars represent mean ± SEM; *N* = 4 biological replicates per species. Differences from the no-odorant control were analyzed by one-way ANOVA followed by Dunnett’s multiple-comparisons test. *P < 0.05, **P < 0.01, ***P < 0.001, ****P < 0.0001.

To identify candidate volatile compounds underlying the species-selective behavioral effects of *P. occidentalis*, individual VOCs from the *P. occidentalis* headspace were tested in cafeteria-style assays with *D. suzukii* and *D. melanogaster* (Fig. 4A). Behavioral responses varied across compounds (Fig. 4B; Fig. S1). The first round of cafeteria-style assays was inconclusive, as *D. melanogaster* was strongly attracted to phenylethyl alcohol (*P* < 0.0001), and modestly to benzyl alcohol, isoamyl decanoate, and ethyl decanoate, leading to a skewed distribution (Fig. S1). *D. suzukii* was also modestly attracted to compounds such as isoamyl octanoate, ethyl dodecanoate, and phenylethyl alcohol, though with notably weaker responses than those observed in *D. melanogaster*. This resulted in a few insects entering the control group. Consequently, we removed these attractive compounds and conducted another cafeteria-style assay (Fig. 4B), which revealed repellent compounds. Benzyl alcohol (*P* < 0.0001) and 2,2-dimethyl-3-hexanone (*P* < 0.0001) were significantly attractive to *D. melanogaster*. In contrast, benzyl alcohol exhibited significantly repellent responses (*P* = 0.0442), and 2,2-dimethyl-3-hexanone was modestly repellent in *D. suzukii*. 3-Ethyl-2,5-dimethylpyrazine elicited modest repellency in *D. suzukii* while remaining behaviorally neutral in *D. melanogaster*. Ethyl decanoate (*P* = 0.0044) and ethyl heptanoate (*P* = 0.0147) were significantly attractive to *D. suzukii* while eliciting a significantly repellent response in *D. melanogaster* (ethyl decanoate, *P* = 0.0396; ethyl heptanoate, *P* = 0.0003). Isoamyl butanoate, benzaldehyde, and 2-phenyl-1-propanal elicited a repellent response in both species (*D. melanogaster*: isoamyl butanoate, *P* < 0.0001; benzaldehyde, *P* = 0.0003; 2-phenyl-1-propanal, *P* = 0.0003; *D. suzukii*: isoamyl butanoate, *P* = 0.0442; 2-phenyl-1-propanal, *P* = 0.0442).

### *P. occidentalis*-emitted repellents outperform standard repellents of *D. suzukii*

**Fig 5:**
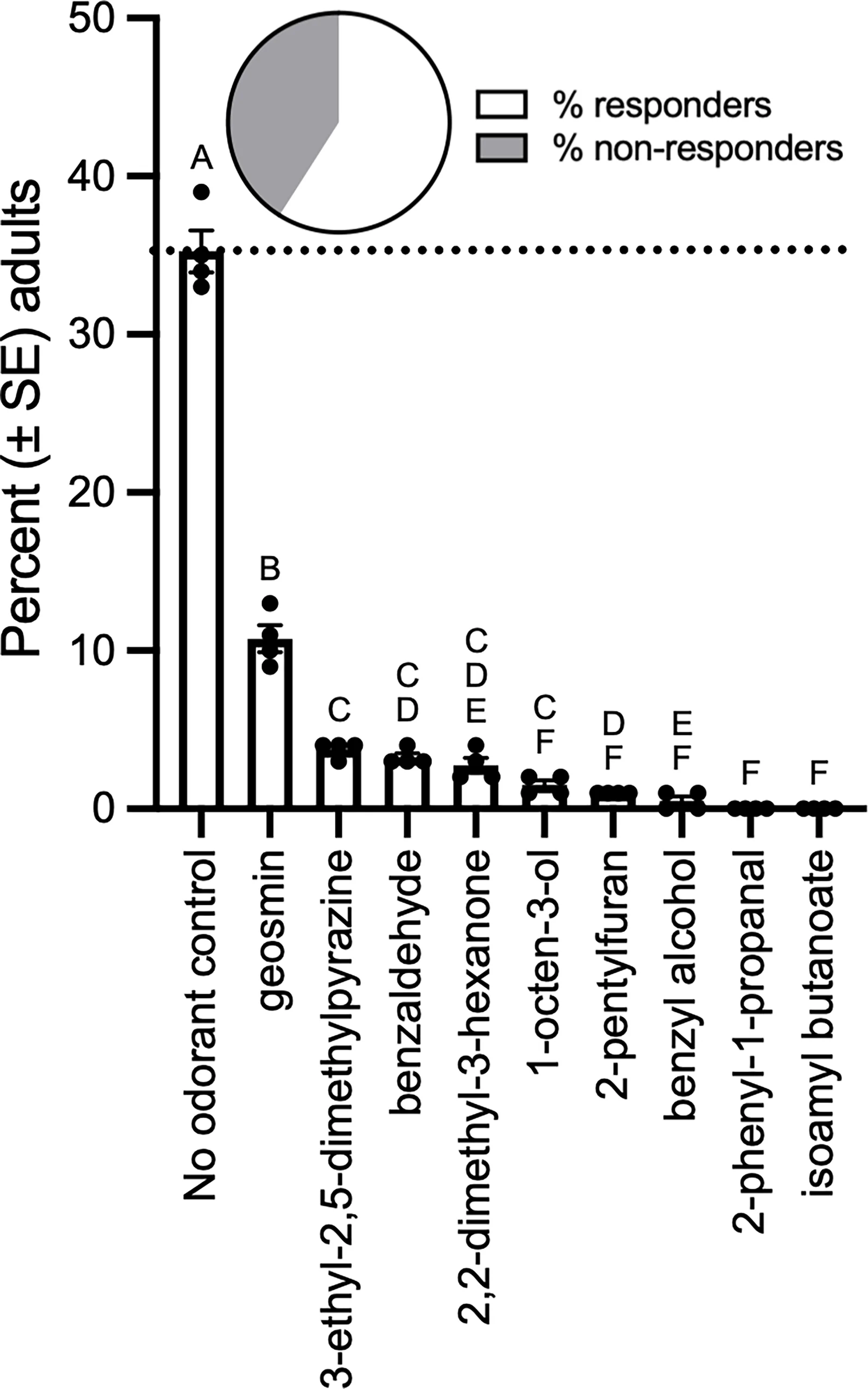
*P. occidentalis*-derived repellents outperform established *D. suzukii* repellents. Percent of *D. suzukii* captured in cafeteria-style traps containing *P. occidentalis*-derived candidate repellents (3-ethyl-2,5-dimethylpyrazine, 2,2-dimethyl-3-hexanone, benzaldehyde, benzyl alcohol, 2-phenyl-1-propanal, isoamyl butanoate) versus established *D. suzukii* repellents (2-pentylfuran, 1-octen-3-ol, geosmin) and a no-odorant control. Calculated as the number of flies in each trap divided by the 300 flies released per cage. Bars represent mean ± SEM; *N* = 4 biological replicates. Differences among treatments were analyzed by one-way ANOVA followed by Tukey’s multiple-comparisons test. Compact letter displays denote statistically distinct groups (*P* < 0.05); bars sharing a letter are not significantly different.

To assess the relative efficacy of *P. occidentalis*-derived repellents, the six most promising compounds from the cafeteria-style assays—3-ethyl-2,5-dimethylpyrazine, 2,2-dimethyl-3-hexanone, benzaldehyde, benzyl alcohol, 2-phenyl-1-propanal, and isoamyl butanoate—were compared in cafeteria-style bioassays with established *D. suzukii* repellents: 2-pentylfuran (10), 1-octen-3-ol (12), and geosmin (24). All compounds reduced *D. suzukii* capture relative to the no-odorant control (all compounds, *P* < 0.0001) (Fig. 5). The *P. occidentalis*-derived compounds demonstrated superior performance compared to established repellents. Both isoamyl butanoate and 2-phenyl-1-propanal achieved complete repellency, capturing no flies throughout the assay period. Benzyl alcohol also showed strong repellent effects, capturing significantly fewer flies than any of the established repellents (isoamyl butanoate: 2-pentylfuran, *P* = 0.0168; geosmin, *P* < 0.0001; 2-phenyl-1-propanal: geosmin, *P* < 0.0001; benzyl alcohol: geosmin, *P* < 0.0001). Among the known repellents, 2-pentylfuran and 1-octen-3-ol performed as expected, while geosmin showed unexpectedly weak repellency, capturing approximately 10% of released flies, significantly more than the *P. occidentalis* compounds but still less than the no-odorant control (geosmin compared to all compounds, *P* < 0.0001). These results underscore the value of mining natural ecological interactions for semiochemical discovery, as compounds shaped by evolutionary selection pressure appear to exhibit greater efficacy than those identified through synthetic screening.

### *P. occidentalis*-emitted repellents are efficacious over a broad concentration range

**Fig 6:**
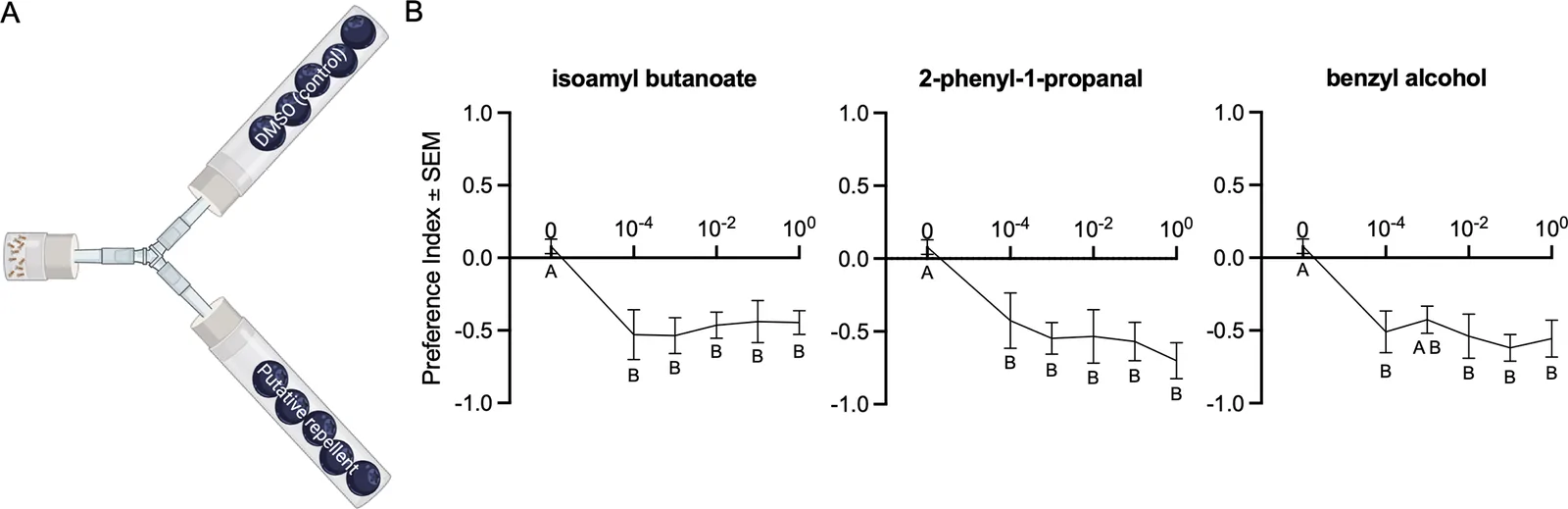
*P. occidentalis*-derived compounds retain repellent activity over a broad concentration range. (**A**) Schematic of the two-choice Y-maze olfactory assay. Ten flies (five female, five male, 7 days old) were introduced into the loading vial and allowed 24 h to choose between a trap containing *P. occidentalis*-derived volatiles (isoamyl butanoate, 2-phenyl-1-propanal, or benzyl alcohol) and a trap containing DMSO (control). (**B**) Preference index (PI) of *D. suzukii* in response to *P. occidentalis*-derived volatiles across concentrations ranging from 0 to neat. PI = [(# in experimental) − (# in control)] / 10; positive values indicate attraction, and negative values indicate avoidance. Bars represent mean ± SEM; *N* = 15 biological replicates per concentration. Differences among concentrations were analyzed using a Kruskal-Wallis test followed by Dunn’s multiple-comparisons test. Different letters above bars indicate statistically significant differences (*P* < 0.05).

To determine the effective concentration range of *P. occidentalis*-derived repellents against *D. suzukii*, dose-response bioassays were conducted using serial dilutions (neat to 10^−5^) of the three most promising compounds—isoamyl butanoate, 2-phenyl-1-propanal, and benzyl alcohol. All three compounds maintained significant repellent activity across the broad concentration range (Fig. 6). Isoamyl butanoate was significant at each dilution from 10^⁰^ to 10^−4^ CFU/mL, with *P* = 0.0272, 0.0409, 0.0208, 0.0073, and 0.0115, respectively. 2-phenyl-1-propanal was significant across the same range, with *P* = 0.0012, 0.0193, 0.0143, 0.0168, and 0.0411. Benzyl alcohol was significant at 10°, 10^−1^, 10^−2^, and 10^−4^ CFU/mL, with *P* = 0.0076, 0.0016, 0.0102, and *P* = 0.0154. The persistence of repellent activity at low concentrations indicates that these *P. occidentalis*-derived compounds are highly potent behavioral modulators. This broad effective range suggests potential for field applications where environmental dilution typically reduces active compound concentrations.

## DISCUSSION

This study identifies *P. occidentalis*, a yeast isolated from *D. melanogaster* oral and fecal secretions, as a species-selective modulator of *D. suzukii* olfactory and reproductive behavior. *P. occidentalis* deters *D. suzukii* and inhibits oviposition while producing the opposite effect in *D. melanogaster*. The convergence of these results around a single isolate recovered from a natural interaction between the two species is consistent with the interpretation that *D. suzukii* reads *P. occidentalis*-derived cues as signals of an unsuitable oviposition substrate. This interpretation rests on laboratory behavioral assays. Whether the avoidance reflects genuinely reduced suitability for offspring and whether it holds under field conditions remain to be established.

In the two-choice olfactory assay, *P. occidentalis* induced a concentration-dependent repellent effect in *D. suzukii*; conversely, *P. occidentalis* was attractive to *D. melanogaster* across the same concentration range. These results fit a growing body of work showing that *D. suzukii* does not respond uniformly to “yeast odor” as a single category. Attraction depends on the yeast species and strain, as well as on culture conditions (18, 21, 29), and even closely related species differ. Some, such as *Hanseniaspora uvarum*, attract *D. suzukii* with high specificity, while others are weak or aversive (21, 30). Whereas *D. melanogaster* is strongly attracted to yeast-driven fermentation cues, *D. suzukii* preferentially exploits ripening fruit and shifts its olfactory preference between fresh-fruit and fermentation cues with physiological state and season (18). Summer morphs favor fruit volatiles and winter morphs fermentation cues (18). The repellent response to *P. occidentalis* we observed is consistent with earlier field observations by Bueno et al. (23), who reported that *Pichia* sp. cultures baited into traps in raspberry plantings captured very few wild *D. suzukii* despite producing detectable headspace volatiles. *Pichia* attraction is species-specific: some species, such as *P. terricola*, are attractive to *D. suzukii* (21). This suggests that the volatile profile of this particular *Pichia* may be intrinsically unattractive or aversive to *D. suzukii* under field conditions, rather than a property of the genus, especially given that other microbial baits in the same study trapped *D. suzukii* in large numbers (23). Our laboratory results extend this pattern by identifying a specific *Pichia* species, *P. occidentalis*, that not only fails to attract *D. suzukii* but actively deters it from ovipositing while simultaneously attracting *D. melanogaster*. The fact that *P. occidentalis* was originally isolated from *D. melanogaster* oral and fecal secretions is consistent with this divergence—the yeast serves as a familiar and attractive microbial signal for *D. melanogaster*, whereas *D. suzukii* processes it as a marker of substrate unsuitability (16). This species-divergent response to a shared yeast is consistent with a role for microbial volatiles in competition between *Drosophila* species: a cue that attracts *D. melanogaster* while deterring *D. suzukii* could help partition the two onto different substrates, echoing evidence that microbes released by competing *Drosophila* repel *D. suzukii* and inhibit its oviposition (15).

*P. occidentalis* exposure significantly reduced survival in both sexes of *D. suzukii*, but the effect was far stronger in males, which showed an 11-day reduction in median survival. In females, the effect was weak and late-emerging, with no change in median survival. Because *P. occidentalis* is not an entomopathogen, this male-biased mortality is unlikely to reflect an infection response. It is more plausibly attributable to non-pathogenic differences between the sexes, such as body size, energetic reserves, or general physiological tolerance to dietary stress. The absence of direct lethality indicates that the effect of *P. occidentalis* on *D. suzukii* is behavioral modification rather than pathogenicity. Indeed, orientation and oviposition assays demonstrated this decoupling. *D. suzukii* females did not avoid fungal-treated substrates during the orientation step, indicating that initial host contact is not deterred, but they strongly avoided fungal substrates during egg laying. *D. melanogaster*, by contrast, preferred *P. occidentalis* substrates for both orientation and oviposition. This decoupling of attraction and oviposition is increasingly recognized in insect-microbe interactions, where microbial cues can differentially regulate host-seeking and reproductive decisions (31). In *D. suzukii* specifically, olfactory and gustatory pathways are known to integrate microbial and fruit-derived signals to optimize oviposition site selection and avoid substrates that may compromise offspring survival (16, 32, 33).

SPME GC-MS identified candidate compounds that may underlie this divergence. Interestingly, benzaldehyde elicited a repellent response in both species, with *D. suzukii* showing slightly greater sensitivity, consistent with prior work showing that benzaldehyde and structurally related aromatic aldehydes function as aversive cues in insects (34), often activating stress-associated olfactory pathways, and that benzaldehyde activates specific olfactory receptor neurons linked to avoidance in *Drosophila* (35).

Two compounds, isoamyl butanoate and 2-phenyl-1-propanal, elicited convergent aversive responses in both *D. suzukii* and *D. melanogaster*. Isoamyl butanoate is notable because esters are, as a class, among the most reliable attractants for drosophilids, mediating host location toward ripening and early-fermenting fruit (36). Its repellency runs counter to the default behavioral valence of its chemical family. This apparent paradox is resolved by a growing body of work showing that ester valence is neither fixed nor uniform across the class: it depends on the specific ester and its concentration. Short-chain acetate esters, such as isoamyl acetate, attract *D. suzukii* at low to moderate concentrations but become repellent at high concentrations, plausibly signaling overripe or microbially advanced substrates that are unsuitable for oviposition (20, 36). More directly, the butanoate esters ethyl butanoate and ethyl (*E*)-but-2-enoate, emitted by *C. fioriniae*-infected blueberries, function as potent, dose-dependent repellents and oviposition deterrents that match or exceed established repellents in efficacy (19, 22). Isoamyl butanoate extends this pattern, indicating that the butanoate moiety in particular can convert an attractive chemical class into an aversive one. That both species share this aversion suggests that it engages a broadly conserved channel or operates at a high effective concentration, rather than exploiting the species-specific olfactory tuning that underlies *P. occidentalis*’s selective effects.

2-Phenyl-1-propanal likewise repelled both species, and although it is largely uncharacterized in insect behavioral studies, its activity is interpretable on structural grounds. As an aromatic aldehyde, it is closely related to benzaldehyde and phenylacetaldehyde, and aromatic aldehydes constitute a recognized class of aversive olfactory cues in *Drosophila* (34, 35). Benzaldehyde drives concentration-dependent avoidance in adult flies, and the receptor Or43a responds to a suite of fruit-derived aromatics that includes benzaldehyde, cyclohexanol, and benzyl alcohol (35, 37). The convergent aversion we observed to benzaldehyde, 2-phenyl-1-propanal, and benzyl alcohol is consistent with these aromatic compounds recruiting overlapping, broadly conserved aversive pathways in both species. Because direct electrophysiological and behavioral data for 2-phenyl-1-propanal are lacking, it represents a priority candidate for targeted electroantennogram and single-sensillum recordings to test whether it activates aromatic-aldehyde-responsive olfactory receptor neurons.

These convergent responses stand in contrast to phenylethyl alcohol, the most abundant compound in the *P. occidentalis* headspace. Phenylethyl alcohol was strongly attractive to *D. melanogaster* and only weakly attractive to *D. suzukii*, a divergence that mirrors the species’ distinct ecological niches. As a canonical yeast- and fermentation-associated volatile, phenylethyl alcohol is a core component of *D. melanogaster* attractant blends. Yeast-derived volatiles, rather than fruit volatiles, are the primary drivers of *D. melanogaster* attraction, oviposition, and larval development (20, 38). *D. suzukii*, by contrast, is specialized on ripening fruit and is comparatively less responsive to fermentation cues, particularly at the high concentrations characteristic of advanced microbial activity; direct comparisons confirm that *D. melanogaster* prefers higher concentrations of phenylethyl alcohol than does *D. suzukii* (39). The behavioral implication is that abundance in the volatile blend does not predict behavioral potency across species: the single most abundant compound in the *P. occidentalis* headspace is a strong attractant for the non-target species and a weak cue for the target pest. This decoupling reinforces the interpretation that *P. occidentalis*’s species-selectivity arises not from any single dominant volatile but from the differential tuning of the two species’ olfactory systems to a shared chemical environment. Individual minor constituents, not the most abundant ones, carry the ecologically decisive signals.

Among these minor constituents, ethyl heptanoate exhibits the opposite species bias to phenylethyl alcohol. Ethyl heptanoate was significantly attractive to *D. suzukii* while eliciting a repellent response in *D. melanogaster*. This polarity opposes that of phenylethyl alcohol and aromatic repellents. This reversal is interpretable through the divergent evolution of ester perception in the two species. *D. suzukii* has extensively remodeled its odorant-receptor repertoire relative to *D. melanogaster*, with duplications and positive selection among receptors tuned to short-chain esters and loss or functional shift of receptors tuned to fermentation-associated volatiles (17, 40). At the level of individual sensory neurons, the *D. melanogaster* ab3A neuron (expressing Or22a) responds strongly to medium-chain esters such as ethyl and methyl hexanoate. In contrast, the cognate *D. suzukii* neuron has lost this high sensitivity and instead acquired increased responsiveness to other esters, consistent with the species’ ecological shift away from fermenting substrates toward ripening fruit (17, 40). Ethyl heptanoate belongs to this same medium-chain ethyl ester class, and its opposing valence across the two species is what the remodeling of these ester-detecting receptors would predict. The concurrent observation that ethyl decanoate produced the same reverse-polarity response reinforces this interpretation, indicating that the divergence is a property of the medium- to long-chain ethyl ester family rather than of a single compound.

A third and distinct pattern emerged for 3-ethyl-2,5-dimethylpyrazine, which elicited significant repellency in *D. suzukii* while remaining behaviorally neutral in *D. melanogaster*. 3-Ethyl-2,5-dimethylpyrazine is the only compound we identified with a strictly *D. suzukii*-selective aversive profile. Alkylpyrazines are nitrogen-containing heterocycles characteristically associated with microbial metabolism, particularly in bacteria, and they function broadly across insects as semiochemicals. Alkylpyrazines serve as resource- and oviposition-location cues, alarm pheromones, and warning signals (41, 42). Their behavioral valence is strongly species- and concentration-dependent. The same alkylpyrazines that act as oviposition attractants for some flies, such as *Rhagoletis pomonella* Walsh and *Anopheles gambiae* Giles, function as aversive or warning cues in other contexts (42). The *D. suzukii*-specific repellency we observed is consistent with this species’ tendency to interpret cues of advanced microbial colonization as markers of substrate unsuitability.

In contrast, the more fermentation-tolerant *D. melanogaster* registers the same compound as neutral. Because alkylpyrazines appear to engage broadly tuned olfactory receptors conserved across diverse insects (41, 42), the basis of this selectivity, and whether *D. suzukii* possesses a dedicated pyrazine-responsive channel, is a clear target for electroantennogram and single-sensillum recording. From an applied standpoint, a volatile that repels the pest while leaving a closely related non-target species unaffected represents the most desirable “push” profile among the compounds we identified. 3-Ethyl-2,5-dimethylpyrazine, therefore, merits particular attention as a candidate species-selective oviposition deterrent.

This reversal also carries distinct applied significance. Whereas the repellent compounds offer a means to push *D. suzukii* away from a protected resource, a compound that is attractive to the pest yet repellent to a non-target relative is well-suited to serve as the complementary “pull” in a stimulo-deterrent diversion or push-pull framework (43), drawing *D. suzukii* into traps or sacrificial zones while sparing *D. melanogaster*. Sparing the non-target relative is not merely incidental. Because microbially mediated competition from *D. melanogaster* can itself suppress *D. suzukii* (15), preserving the competitor may confer an additional indirect measure of control. Together, the species-divergent compounds in the *P. occidentalis* headspace (i.e., phenylethyl alcohol biased toward *D. melanogaster*; ethyl heptanoate and ethyl decanoate biased toward *D. suzukii*; the aromatic aldehydes aversive to both; and 3-ethyl-2,5-dimethylpyrazine aversive to *D. suzukii* alone) constitute a mosaic of opposing signals rather than a single uniform cue. This heterogeneity is itself the central finding: it implies that the species-selectivity of *P. occidentalis* emerges from the differential tuning of two olfactory systems to a shared volatile blend, such that the same yeast can be read as an attractive host marker by one species and as an unsuitable substrate signal by the other.

Situated within an IPM program, these compounds map onto complementary roles: the *D. suzukii*-selective repellents (i.e., isoamyl butanoate, 2-phenyl-1-propanal, benzyl alcohol, and 3-ethyl-2,5-dimethylpyrazine) could be deployed at the crop as oviposition deterrents to protect ripening fruit. At the same time, a pest-attractive, non-target-repellent cue such as ethyl heptanoate could be paired with existing attractant-based traps to divert flies toward monitoring or removal (6, 43). Because these are volatile behavioral cues rather than contact insecticides, they act at a different point in the pest’s decision-making than conventional controls. They could reduce spray frequency while sparing closely related non-target species. Integration with monitoring and targeted insecticide use, rather than its replacement, is the most realistic near-term deployment.

The three established repellents do not share a common mode of action. Geosmin acts through the narrowly tuned labeled line Or56a (24), whereas 1-octen-3-ol and 2-pentylfuran engage more broadly tuned receptors (19, 22). Geosmin’s weak performance in our cafeteria assay (capturing roughly 10% of released flies despite being the most potent single repellent described in *Drosophila*) is readily explained. Geosmin is far less volatile than the other compounds, with a vapor pressure three orders of magnitude below that of benzaldehyde. At equal volume, geosmin generated a lower headspace concentration (24). Moreover, because each trap presented attractive blueberry volatiles, a low-volatility cue that acts mainly by suppressing attraction is the one most easily overshadowed (44). That these compounds outperform established repellents may reflect their evolutionary history as mediators of interspecific competition, in which activity has been selected in ways that synthetic screening may not replicate.

Viewed this way, the broad effective concentration range of the *P. occidentalis*-derived compounds (Fig. 6) is mechanistically consistent with this interpretation: compounds that retain aversive activity across several orders of magnitude of dilution are those most likely to remain above the behavioral threshold and sustain an unfavorable attractant-to-repellent ratio, even as environmental dilution reduces their concentration. This property directly addresses the principal weakness of geosmin in an applied setting. Nevertheless, the comparison reported here is specific to a 24 h trap-capture format with unnormalized emission rates, and the relative rankings could shift under conditions in which release rates are matched. Confirmation will require semi-field and field evaluations using controlled, normalized emission rates, as has been done for ester repellents of *D. suzukii* in blueberry plantings (22), to establish whether the laboratory advantage of these yeast-derived compounds translates to protection of fruit under realistic dispersal and competition conditions.

The headspace profile of our *P. occidentalis* isolate differed substantially from the profile previously reported for strain KKP 3004 (26): our isolate emitted phenylethyl alcohol as the overwhelmingly dominant volatile (~90–97% of total peak area), with comparatively few of the medium-chain ethyl and isoamyl esters, aromatic aldehydes, and terpenes that characterized KKP 3004. This divergence is expected given the biological and analytical differences between the studies. Phenylethyl alcohol is produced from L-phenylalanine through the Ehrlich pathway, in which phenylacetaldehyde, also detected in our headspace, is the immediate biosynthetic precursor (45). The prominence of both compounds indicates strong Ehrlich pathway flux in our isolate under our culture conditions. Because flux through this pathway is strongly governed by nitrogen source and medium composition, with phenylalanine-rich or nitrogen-limited conditions favoring phenylethyl alcohol accumulation and ammonium repressing it (45), differences in growth substrate between the two studies (yeast extract peptone dextrose versus the glucose-yeast-extract medium used by Choińska et al. [26]) readily account for a shift from ester-dominated to phenylethyl alcohol-dominated emission. Moreover, yeast volatile production is highly strain-specific and sensitive to culture conditions (28, 45). Our isolate and KKP 3004 also originate from different hosts and geographic regions. The two studies also used different SPME fiber chemistries and reported semiquantitative relative abundances rather than absolute concentrations. The dominance of phenylethyl alcohol in our profile compresses the relative abundance of all minor constituents, such that low-abundance esters present in the headspace may fall below detection. For these reasons, the two profiles are best regarded as qualitatively consistent strain- and condition-specific variants rather than discrepant, and the detection of phenylethyl alcohol and acetophenone in both supports the identity of our isolate while underscoring why we drew the behavioral candidate panel from the published profile.

Together, these findings indicate that *P. occidentalis* does not primarily function as a lethal agent against *D. suzukii*, but rather as a modulator of olfactory and reproductive behavior. The observed aversion to oviposition, despite maintained proximity, suggests that fungal-derived cues signal reduced suitability of the substrate for offspring development, which aligns with recent work demonstrating that microbial community composition can shape oviposition decisions in Drosophilids by altering both volatile profiles and substrate quality (15, 16, 31). From an applied perspective, this distinction is critical. Because female *D. suzukii* are the primary drivers of crop damage through egg deposition in intact fruit (3, 4), strategies that disrupt oviposition behavior may be more effective than those relying on mortality alone. Even partial deterrence of oviposition could reduce larval infestation rates and subsequent population growth (11, 12). *P. occidentalis* and its derived volatiles, particularly phenylethyl alcohol, are therefore better positioned as components of behavior-based management strategies, such as push-pull systems or oviposition deterrents, than as conventional biocontrol agents relying on direct pathogenicity. The species-selectivity of the response is a particular strength of this approach, since it suggests a route to repelling *D. suzukii* and deterring oviposition without disrupting closely related non-target *Drosophila*.

The environmental context of these compounds also suggests interesting parallels with other naturally occurring pest repellents and oviposition deterrents. The isolation of *P. occidentalis* from *D. melanogaster* oral and fecal secretions, combined with its selective effects against *D. suzukii*, points toward a potential role in chemical niche partitioning between competing *Drosophila* species. This finding aligns with emerging evidence that microbial communities actively shape insect behavior and may drive ecological specialization through volatile-mediated mechanisms (21, 46, 47).

However, whether these chemical interactions operate similarly in naturalistic contexts remains to be established. Using individual compounds does not recapitulate the complexity of natural volatile blends, in which synergistic or antagonistic interactions among compounds can significantly alter behavioral outcomes. Because our headspace analysis reports relative rather than absolute abundances, we did not quantify how the neat compound concentrations used for screening compare with the amounts emitted by the *P. occidentalis* biofilm. Moreover, the cafeteria-style assays used four replicate cages per treatment, which is well-suited to detecting the large effects underlying our primary conclusions but limits the power to resolve smaller differences among individual compounds. Future work should evaluate candidate compounds in combination, validate their effects under field conditions, and assess the stability and scalability of candidate yeast-derived repellents for agricultural applications.

Despite these limitations, the present study expands current understanding of how environmental fungi influence *D. suzukii* behavior. By tracing a species-selective oviposition deterrent from an unexpected colony collapse to a candidate repellent compound, it identifies *P. occidentalis* and isoamyl butanoate, 2-phenyl-1-propanal, and benzyl alcohol as promising starting points for behavior-based, species-selective approaches to *D. suzukii* management.

## MATERIALS AND METHODS

### Isolation and identification of *P. occidentalis*

*P. occidentalis* was isolated from *D. melanogaster* oral and fecal secretions on *D. suzukii* diet and streaked onto yeast extract peptone dextrose (YPD) agar plates (1% wt/vol yeast extract, 2% peptone, 2% dextrose, 2% agar). YPD broth was prepared identically without agar. Plates were incubated at 30°C overnight, and isolated colonies were transferred to YPD broth. DNA was isolated using the Monarch Spin gDNA Extraction Kit following the sample tissue lysis protocol (New England Biolabs, Ipswich, MA). Briefly, a couple of loopfuls of culture were scraped off the plate and suspended in a 2 mL tube containing two 5 mm ball bearings, 200 µL of Tissue Lysis buffer, and 10 µL of Proteinase K. The sample was homogenized in a Qiagen Tissue Lyser II (Qiagen, Germantown, MD) for 90 s at 30 Hz. After homogenization, the sample was incubated at 56°C for 1 h with gentle mixing by inversion every 5–10 minutes. After 1 h, 3 µL of RNase A was added, and the sample was returned to the 56°C incubator for 5 min. The genomic DNA binding and elution steps were performed according to the protocol (New England Biolabs). The extracted DNA was used for PCR. Primers used were ITS1 (TCCGTAGGTGAACCTGCGG) and ITS4 (TCCTCCGCTTATTGATATGC). Each 25 µL reaction contained 0.5 µL of each primer (10 µM stock), 5 µL of Taq 5X Master Mix (New England Biolabs), 1 µL of template DNA (about 50 ng), and water to a final volume of 25 µL. Cycling parameters consisted of initial denaturation (95°C for 30 s) followed by 30 cycles of 95°C for 30 s, 55°C for 30 s, 68°C for 1 min, and a final extension at 68°C for 5 min. The reaction products were analyzed on a Qiaxcel to confirm amplification and the expected fragment size (about 480 bp). The fragments were purified using ExoSAP-It Express (Thermo Fisher Scientific, Waltham, MA) and sequenced using the BigDye 3.1 Terminator Cycle Sequencing Kit (Thermo Fisher Scientific). After cycle sequencing, the reaction was cleaned using the BigDye Xterminator Purification Kit (Thermo Fisher), and the sequencing reactions were run on the ABI 3500 Genetic Analyzer (Thermo Fisher). Sequence data were compared to those in GenBank using BLASTN. Results indicated that the yeast belonged to the genus *Pichia*. To further identify the isolated yeast to species, the isolated DNA was amplified by PCR as above, using the primers NL1 (GCATATCAATAAGCGGAGGAAAAG) and NL4 (GGTCCGTGTTTCAAGACGG). This primer set amplifies the D1–D2 regionof the large-subunit RNA gene and is useful in identifying yeast cultures to species (48). Reactions run on Qiaxcel showed amplicons of about 680 bp. Sequencing was as above. Sequence data were used for BLASTN. Alignments were done in Mega 11 (49).

### *D. suzukii* and *D. melanogaster* colony maintenance

*D. suzukii* specimens were donated by the New Jersey Department of Agriculture. *D. suzukii* was collected in 2013 from wild-collected flies and supplemented with additional wild genetic material every 1–2 years. *D. suzukii* were maintained in the laboratory on a standard diet prepared with 45 g agar, 125 g cornmeal, 200 g sugar, 70 g nutritional yeast (deactivated *Saccharomyces cerevisiae*), and 3.8 L dH₂O. Fungal inhibitors were added: 17.7 mL of 1 M propionic acid, 3.3 g of methyl paraben, and 33.3 mL of 95% ethanol. *D. melanogaster* (Canton-S; RRID:BDSC_64349) were maintained on Nutri-Fly Bloomington Formulation (Genesee Scientific) prepared according to the manufacturer’s instructions. Propionic acid (Sigma; final 0.3% vol/vol) was added as a fungal inhibitor. All food was autoclaved and cooled to room temperature before preservatives were added, then distributed into narrow polypropylene vials (Genesee Scientific) within a biosafety cabinet. Unless otherwise stated, flies were maintained at 25°C, 60% relative humidity (RH), under a 16 h light/8 h dark (16L:8D) cycle. Populations were age-matched and density-controlled by seeding ~30 adults per vial for oviposition; adults were removed after 24–48 h to standardize larval density. Experimental and control treatments were run concurrently to control for daily variation in fly activity and blueberry emissions. Because female *D. suzukii* cause crop damage through oviposition in intact fruit, oviposition and orientation assays were conducted with mated females; the two-choice olfactory and cafeteria assays used mixed-sex groups scored as pooled responses, and the longevity assay was analyzed separately by sex. *D. suzukii* and *D. melanogaster* are invertebrates and are not subject to IACUC oversight under U.S. federal regulations (Animal Welfare Act, 9 CFR Parts 1–3).

### Two-choice olfactory assay

Flies were starved for 24 h at 25°C before testing. The Y-maze was assembled by connecting a Y-shaped connector to two glass trap vials and one loading vial via trimmed pipette tips through foam stoppers (Fig. 1A), as described by Simonnet et al. (50). Each trap vial was loaded with either 20 µL of *P. occidentalis* or YPD (control), corresponding to ~ 2 × 10^7^ cells. Ten flies (five female, five male, 7 days old) were introduced into the loading vial using brief (<5 min) ice anesthesia and allowed 24 h to make their choice in the dark at 25°C, with maze orientation alternated throughout. The apparatus was cleaned by overnight soaking in detergent (Alconox), followed by rinses with tap water and deionized water. Preference index (PI) was calculated as [(# in experimental) − (# in control)] / 10.

### Adult longevity assay

Adult survival when exposed to *P. occidentalis* was assessed using a 96-well plate survival assay (Fig. 2A), adapted from Linford et al. (51). Individual flies were housed singly in wells of a 1.0 mL deep-well plate (Biologix) containing 500 µL standard *D. suzukii* diet, either supplemented with *P. occidentalis* (experimental) or YPD (control). The diet surface was inoculated using spread plate technique and incubated at 30°C overnight. Individual wells were randomized to blind the experimenter to treatment conditions. Flies were transferred to fresh 96-well plates containing treated food every day without anesthesia, using a pooter through a small notch cut in the lid above each well. Each well was covered with breathable rayon sealing film (Axygen) after transfer. At each transfer, dead flies were counted and recorded. Any flies lost to escape or accidental death were recorded on the day of exit and excluded from mortality counts. Transfers continued until the last survivor died. For each species, sex, and condition, 24 flies were monitored until death.

### Oviposition and orientation assay

The oviposition and orientation assay was adapted from Sato et al. (16) to evaluate behavioral responses to *P. occidentalis*. Adult female *D. suzukii* and *D. melanogaster* were placed on 25 mL food plates under three treatment conditions: fully YPD (control), fully inoculated with *P. occidentalis* (control), or split plates with one half streaked with YPD and one half inoculated with *P. occidentalis* (Fig. 3A). For each trial, 20 females were mated for 3 days before being introduced to the plates using brief ice anesthesia. Fly distribution was recorded 15 min after introduction to capture initial orientation preference before the onset of substantial oviposition activity, providing a positional readout distinct from subsequent egg-laying decisions. Flies were then allowed to oviposit for 3 h, after which egg counts were recorded for each treated region.

### Selection of candidate volatile compounds

Headspace volatiles were collected by solid-phase microextraction (SPME) using two complementary fiber chemistries to broaden the range of captured analytes: a 100 µm polydimethylsiloxane fiber (PDMS; fused silica/stainless steel) and an 85 µm carboxen/polydimethylsiloxane fiber (CAR/PDMS; StableFlex/stainless steel) (Supelco, Bellefonte, PA, USA). The PDMS coating preferentially retains less volatile, non-polar compounds, whereas the microporous CAR/PDMS coating favors small, highly volatile analytes; sampling with both fibers therefore captured a wider volatility range than either alone. After 5 days of incubation, each headspace was extracted separately with each fiber: the fiber was inserted through a small hole in the Parafilm seal, exposed to the headspace vapor for 45 min at room temperature, then withdrawn and immediately thermally desorbed for 30 s in the injection port, operated in splitless mode at 170°C, of a Varian 3800 gas chromatograph (Walnut Creek, CA, USA) coupled to a Varian Saturn 2000 ion-trap mass spectrometer.

Chromatographic separation was performed on a Restek Rxi-5Sil MS crossbond capillary column (30 m × 0.25 mm i.d., 0.25 µm film; stationary-phase polarity equivalent to 5% diphenyl/95% dimethyl polysiloxane; Restek, Bellefonte, PA, USA) using helium as the carrier gas at 1 mL/min. The oven program began at 35 °C (held 1 min), ramped at 10°C/min to 220°C, and held at 220°C for 10 min, for a total run time of 29.5 min. The manifold, GC-MS interface, and ion trap were maintained at 50°C, 250°C, and 180°C, respectively. Mass spectra were acquired in electron-impact mode (70 eV) over a scan range of m/z 35–200. Volatile compounds were tentatively identified by matching their mass spectra against the NIST 98 MS library.

Candidate volatiles for behavioral testing were also selected from the published *P. occidentalis* headspace VOC profile reported by Choińska et al. (26), which characterized strain KKP 3004 by SPME GC-MS. Compounds reported in either the present headspace analysis or the published profile of Choińska et al. (26) that were commercially available were included in the behavioral evaluation.

### Multiple-choice (cafeteria-style) olfactory assay

Cafeteria-style olfactory assays were conducted to evaluate the responses of adult *D. suzukii* and *D. melanogaster* to individual *P. occidentalis*-derived volatiles, following methods adapted from Rering et al. (19). Assays were performed in 14″ × 14″ × 14″ netted cages containing traps arranged equidistantly in a circular pattern around a central water source (Fig. 4A). Each trap consisted of a 50 mL fly food tube sealed with Parafilm and fitted with a 4 mm hole for fly entry. Traps contained 5 g of organic blueberries that had been sterilized with 3% bleach and rinsed with distilled water until the bleach odor dissipated. Control traps contained only blueberries, while experimental traps additionally included one volatile compound (50 µL, neat) applied to a cotton substrate inside the tube. Three hundred adult flies (1:1 male:female ratio) were collected by pooter into an empty fly vial. Following 2 h of starvation, the vial was placed in the center of the cage to release flies. Another vial containing cotton soaked in deionized water was placed at the center of the arena to prevent desiccation. After 24 h, the number of flies captured in each trap was recorded. Each experiment was replicated four times per species.

### Data analysis

For two-choice olfactory and oviposition/orientation assays, differences among treatment groups were analyzed using a Kruskal-Wallis test followed by Dunn’s multiple-comparisons test. For the cafeteria-style behavioral assays comparing individual volatiles against the no-odorant control, differences were analyzed using a one-way ANOVA followed by Dunnett’s multiple-comparison test against the control. For the cafeteria-style behavioral assay ranking candidate repellents against established repellents, differences among treatments were analyzed using a one-way ANOVA followed by Tukey’s multiple-comparison test, with letters indicating statistically distinct groups (*P* < 0.05). For dose-response cafeteria-style assays, the effect of concentration on fly capture was assessed using a one-way ANOVA followed by Dunnett’s multiple-comparison test against the no-odorant control. For adult longevity assays, Kaplan-Meier survival curves were generated, and differences between groups were evaluated using the Mantel-Cox (log-rank) test. Hazard ratios and 95% confidence intervals were calculated using a Cox proportional hazards model. All statistical analyses were performed in GraphPad Prism (RRID:SCR_002798).

## Data Availability

The raw data supporting the conclusions of this article will be made available by the authors on request.

## SUPPLEMENTAL MATERIAL

Supplemental material aem.01135-26-s0001.docx Table S1 and Fig. S1.

ASM does not own the copyrights to Supplemental Material that may be linked to, or accessed through, an article. The authors have granted ASM a non-exclusive, world-wide license to publish the Supplemental Material files. Please contact the corresponding author directly for reuse.
