# Supplementary material for: Pichia occidentalis volatiles elicit divergent behavioral responses in Drosophila suzukii and Drosophila melanogaster
Source: Appl Environ Microbiol. 2026 Oct 1;92(10):e01135-26. doi: 10.1128/aem.01135-26 (PMC13689338; doi:10.1128/aem.01135-26)
Supplement: Supplementary file 1 [file aem.01135-26-s0001.docx]

# Supplementary Materials

**Supplementary Table**

**Table S1.** Volatile organic compounds identified in the headspace of the *P. occidentalis* isolate by SPME GC-MS, sampled with two fibers (100 µm PDMS and 85 µm CAR/PDMS) across three independent trials per fiber. Values are relative percentages of the total peak area for each trial and the mean of the three trials. Blank cells indicate the compound was not detected. Compounds are listed in elution order, with retention indices calculated on a Restek Rxi-5Sil MS column. Identifications are tentative and based on comparisons of mass spectra with the NIST 98 library. One compound (RI 1589) remained unidentified.

|  | **Compound** | **Retention index (RI)** | **PDMS** | | | | **CAR/PDMS** | | | |
| --- | --- | --- | --- | --- | --- | --- | --- | --- | --- | --- |
|  |  |  | **Trial 1** | **Trial 2** | **Trial 3** | **Avg** | **Trial 1** | **Trial 2** | **Trial 3** | **Avg** |
| 4.289 | Isopentyl formate | 788 |  |  |  |  | 0.54 | 0.44 | 0.19 | **0.39** |
| 4.342 | 1-octene | 791 |  |  |  |  | 1.12 | 0.86 | 0.78 | **0.92** |
| 4.454 | 4E-octene | 798 |  |  |  |  | 0.32 | 0.16 | 0.15 | **0.21** |
| 5.0545 | Isovaleric acid | 834 | 0.55 | 0.14 |  | **0.35** | 1.31 | 0.16 | 0.11 | **0.53** |
| 5.18 | Isopropyl butanoate | 843 |  |  |  |  | 4.71 | 1.88 | 0.97 | **2.52** |
| 5.208 | 2-methylbutanoic acid | 843 | 0.29 | 0.13 | 0.02 | **0.15** |  |  |  |  |
| 5.317 | Ethyl isovalerate | 850 |  |  |  |  | 4.25 | 1.22 | 0.75 | **2.07** |
| 5.457 | 4-pentenoic acid | 858 |  |  |  |  | 0.98 | 0.89 | 0.79 | **0.89** |
| 5.956 | 2-heptanone | 888 | 0.06 | 0.06 | 0.06 | **0.06** | 0.44 | 0.59 | 0.55 | **0.53** |
| 6.857 | 3-methyl-1-butanol | 941 |  |  |  |  | 0.26 | 0.23 | 0.27 | **0.25** |
| 7.062 | 6-methylheptan-2-ol | 953 |  |  |  |  | 0.39 | 0.31 | 0.29 | **0.33** |
| 7.692 | 3-methylbutyl acetate | 990 |  |  |  |  | 0.50 | 0.59 | 0.22 | **0.44** |
| 8.956 | Acetophenone | 1067 | 0.05 | 0.05 |  | **0.05** |  |  |  |  |
| 9.157 | 5Z-octenol | 1079 | 0.05 | 0.09 | 0.09 | **0.08** |  |  |  |  |
| 9.31 | 2-nonanone | 1089 | 0.26 | 0.37 | 0.42 | **0.35** | 0.00 | 0.30 | 0.27 | **0.19** |
| 9.484 | 2-nonanol | 1099 | 0.08 | 0.06 | 0.07 | **0.07** |  |  |  |  |
| 9.705 | Phenylethyl alcohol | 1113 | 96.92 | 96.74 | 97.02 | **96.89** | 84.30 | 92.38 | 94.51 | **90.40** |
| 12.146 | Phenylacetaldehyde | 1275 | 1.51 | 2.10 | 1.95 | **1.85** |  |  |  |  |
| 12.362 | 2-undecanone | 1290 | 0.08 | 0.14 | 0.18 | **0.13** |  |  |  |  |
| 12.818 | Phenyl ethyl oxyacetaldehyde | 1324 |  |  |  |  | 0.80 | 0.23 |  | **0.52** |
| 16.243 | unknown | 1589 | 0.14 | 0.11 | 0.19 | **0.14** |  |  |  |  |

**Supplementary Figure**

**Figure S1. Initial cafeteria-style assay reveals strongly attractive *P. occidentalis*-derived volatiles that obscure repellent effects.** Percent of *D. suzukii* (left) and *D. melanogaster* (right) flies captured per trap after 24 h of exposure to individual *P. occidentalis*-derived volatiles in a cafeteria-style assay. Calculated as the number of flies in each trap divided by the 300 flies released per cage. *D. melanogaster* showed strong attraction to phenylethyl alcohol, benzyl alcohol, and isoamyl decanoate, while *D. suzukii* was weakly attracted to isoamyl octanoate, ethyl dodecanoate, and phenylethyl alcohol, resulting in skewed distributions with few flies entering control or other experimental traps. These strongly attractive compounds were excluded from subsequent assays to enable resolution of repellent responses. Bars represent mean ± SEM; *N* = 4 biological replicates per species. Differences from the no-odorant control were analyzed by one-way ANOVA followed by Dunnett’s multiple comparisons test. **p* < 0.05, ***p* < 0.01, ****p* < 0.001, *****p* < 0.0001.
